# Fenofibrate Ameliorated Systemic and Retinal Inflammation and Modulated Gut Microbiota in High-Fat Diet-Induced Mice

**DOI:** 10.3389/fcimb.2022.839592

**Published:** 2022-06-02

**Authors:** Xue Wang, Chaofeng Yu, Xiaomei Liu, Jiasong Yang, Yuliang Feng, Yajun Wu, Yali Xu, Yihua Zhu, Wensheng Li

**Affiliations:** ^1^ Aier School of Ophthalmology, Central South University, Changsha, China; ^2^ University of Science and Technology of China, Suzhou Institute of Biomedical Engineering and Technology, Suzhou, China; ^3^ Department of Ophthalmology, Shanghai Aier Eye Hospital, Shanghai, China; ^4^ Department of Ophthalmology, First Affiliated Hospital of Fujian Medical University, Fuzhou, China

**Keywords:** gut microbiota, high-fat diet (HFD), inflammation, fenofibrate, retina

## Abstract

Fenofibrate, as a lipid-lowering drug, has been reported to have a protective effect on the retina independent with plasma lipid levels. This study aimed to investigate that the ameliorative effects of fenofibrate on systemic and retinal inflammation, as well as gut microbiota dysbiosis in high-fat diet (HFD)-induced mice. C57BL/6J mice were randomly allocated into four groups: standard diet (SD) group; HFD group; SD plus fenofibrate (SD_ Fe) group; HFD plus fenofibrate (HFD_ Fe) group. After successfully establishing models (5 months), indicators associated with lipid, gut barrier, inflammation and gut microbiota were investigated. Our results showed that supplementing the HFD with fenofibrate decreased body weight gain, alleviated dyslipidemia and reversed the downregulation of short-chain fatty acid (SCFAs) in serum, retina and feces. Fenofibrate ameliorated intestinal barrier function damage in HFD-induced mice. Fenofibrate coadministration inhibited the levels of inflammatory factor and lipopolysaccharide (LPS) in the serum and attenuated inflammatory response in the retina of HFD-induced mice. Systemic LPS was positively correlated with a series of inflammatory factors in serum and retina, respectively. Fenofibrate supplementation down-regulated the abundances of LPS-associated bacteria in HFD mice, including *Firmicutes* and *Proteobacteria* at the phylum level, *Desulfovibrionaceae* at the family level, as well as *unclassified_ Desulfovibrionaceae*, *Acetatifactor*, *Flavonifractor*, *Oscillibacter* and *Anaerotruncus* at the genus level. However, fenofibrate treatment up-regulated the abundances of SCFA-associated bacteria in HFD mice, including *Bacteroidetes* at the phylum level, *Porphyromonadaceae* at the family level, as well as *unclassified_Porphyromonadaceae*, *Barnesiella*, *Alloprevotella* and *Bifidobacterium* at the genus level. In conclusion, our results confirmed fenofibrate could attenuate HFD-induced systemic and retinal inflammation, accompanying with restoration of intestinal barrier damage and modulation of gut microbiota/metabolites. This work provided an explanation for the ameliorative effects of fenofibrate on HFD-induced systemic and retinal inflammation might be partially related with the modulation of gut microbiota and its metabolites.

## Introduction

Fenofibrate, a peroxisome proliferator–activated receptor alpha (PPARα) agonist, has been approved by the USA Food and Drug Administration (FDA) for the treatment of hypertriglyceridemia, primary hypercholesterolemia, or mixed dyslipidemia (Sidhu and Tripp, 2021). The Fenofibrate Intervention and Event Lowering in Diabetes (FIELD) study showed that fenofibrate treatment reduces the need for laser treatment for diabetic retinopathy, which didn’t seem to be related to plasma lipid levels (Keech et al., 2007). In diabetic models (including streptozotocin-induced rat, *db/db* mice and Akita mice), it has been reported that high glucose could directly induce PPARα down-regulation in the retina (Hu et al., 2013). Moreover, fenofibrate could reverse PPARα-downregulation, in turn inhibit retinal inflammation in type I diabetic rodent models (Abcouwer, 2013). Inconsistent with the above diabetic models, high-fat diet (HFD) consumption might activate PPARα-mediated lipid metabolism because fatty acids are PPARα natural ligands (Forman et al., 1997). However, much less has been understood about the effect of fenofibrate on HFD alone-induced retinal inflammation and its mechanism.

Gut microbiota is characterized by an inter-individual difference because of genetic and environmental factors. Among the environmental factors, dietary habits (such as high-fat diet) play a critical role in regulating the composition of gut microbiota (Proctor et al., 2017). Subsequently, gut microbiota exerts profound influences on digestion, dietary metabolism, endotoxemia, and immune responses of its host (Turnbaugh et al., 2006; Cani et al., 2008; Cerf-Bensussan and Gaboriau-Routhiau, 2010). Retinal health has been reported to be closely related with gut microbiota, and thereby the concept of gut-retina axis is proposed (Rowan et al., 2017; Rinninella et al., 2018). Recent study has demonstrated that gut microbiota dysbiosis could increase the laser-induced choroidal inflammation and neovascularization through inducing increased intestinal permeability and metabolic endotoxemia in HFD (7 weeks) feeding mice (Andriessen et al., 2016). In retinitis pigmentosa (RP) model mice, short-term (2-3 weeks) HFD induced the retinal injury accompanying with the changes in gut microbiome (Kutsyr et al., 2021). However, this association between retina and gut microbiota didn’t be found in HFD alone-induced C57BL/6J mice (Kutsyr et al., 2021). In the current study, we investigated whether the cross-talks exits between gut and retina in long-term (5 months) HFD alone-induced C57BL/6J mice. Moreover, this study aimed to first investigate the relationship between anti-inflammatory effect of fenofibrate administration on the HFD alone-induced retina and the modulation of gut microbiota.

## Methods and Materials

### Animals

Four-week-old C57BL/6J male mice (n=200) were provided by the Shanghai Slack Laboratory Animal Center. The animals were kept under environmentally controlled conditions at a temperature of 22–24°C, relative humidity of 50–60%, and an alternating 12h light/dark cycle circumstance. All the mice took a week for acclimatization and were randomly divided into 4 groups (n=50 mice/group): (a) Standard-fat diet (SD) group: four-week-old mice were fed a SD (10% energy from fat, Research Diets XTCON50J, Jiangsu XIETONG Bioengineering Co., Ltd, Nanjing, Jiangsu, China) for 5 months; (b) High-fat diet (HFD) group: four-week-old mice were fed a HFD (60% energy from fat, Research Diets XTHF60, Jiangsu XIETONG Bioengineering Co., Ltd, Nanjing, Jiangsu, China) for 5 months; (c) SD plus fenofibrate (SD_ Fe) group: four-week-old mice were fed a SD for 5 months, and supplemented with 0.1% w/w fenofibrate (F6020; Sigma-Aldrich, St. Louis, MO, USA) in the last month. (d) HFD plus fenofibrate (HFD_ Fe) group: four-week-old mice were fed a HFD for 5 months, and supplemented with 0.1% w/w fenofibrate in the last month. After successfully establishing models (5 months), body weights were recorded, fecal samples were collected and colonic permeability was detected. Subsequently, mice were sacrificed by cervical dislocation and blood, retina, fat and colonic tissue samples were collected.

### Detection of Lipid Concentration

Blood samples were collected from SD, HFD, SD_ Fe and HFD_ Fe groups by cardiac puncture at the time of sacrifice. Total cholesterol (TC), low-density lipoprotein/very low-density lipoprotein cholesterol (LDL/VLDL-C) and high-density lipoprotein cholesterol (HDL-C) levels in serum were respectively measured enzymatically using a cholesterol assay kit (ab65390; Abcam, Cambridge, MA, USA) in accordance with the manufacturer’s instructions. Total triglyceride (TG) level in serum was determined using a triglyceride assay kit (ab65336; Abcam).

The feces, serum and retina from mice were collected and frozen at −80°C. Acetate acid, propionate acid, butyrate acid, isobutyric acid, isovaleric acid, valeric acid, caproic acid in fecal samples were analyzed using gas chromatography–mass spectrometry (GC–MS) (Hoving et al., 2018). The SCFAs in serum and retina samples were analyzed using liquid chromatography-mass spectrometry/mass spectrometry (LC-MS/MS).

### Colonic Permeability Assay

Mice were injected by oral gavage with 4,000-Da fluorescein isothiocyanate (FITC)-dextran (FD4, #46944, Sigma-Aldrich) by gavage (600 mg/kg body, 50 mg/ml). After 4 h, 120 μL of blood was collected from the tip of the tail vein. Isolated serum was diluted in an equal volume of PBS (pH=7.4) and analyzed for FITC-dextran concentration with a fluorescence spectrophotometer (Thermo Scientific, Waltham, MA, USA) at the excitation wavelength of 485 nm and the emission wave length of 535 nm. Standard curves for calculating the FITC-dextran concentration in the samples were obtained by diluting FITC-dextran in nontreated serum diluted with PBS.

### ELISA Assays

Retina tissue were washed and then homogenized in cold PBS. After centrifugation, the supernatant fluids were collected to measure the protein concentrations using a BCA kit (P0012; Beyotime, Nantong, Jiangsu, China). Interleukin (IL)-1β levels in retina and serum were measured by ELISA kit according to the manufacturer’s instruction (ab100704; Abcam, Cambridge, MA, USA). Retina and serum inflammatory cytokines including Tumor Necrosis Factor-alpha (TNFα) (#430907; Biolegend, San Diego, CA, USA) and Interleukin (IL)-6 (#431307; Biolegend) were measured using ELISA kits. Serum lipopolysaccharide (LPS) concentration was determined using a kit (CSB-E13066m; Cusabio, Houston, TX, USA).

### Immunofluorescence Staining

Enucleated eyes were immediately fixed in 4% paraformaldehyde for 12 h at 4°C and cornea and lens were removed. The eye cups were then sequentially transferred to 10%, 20%, 30% sucrose for cryopreservation and embedded in OCT compound. Cryosections were obtained at a thickness of 12 μm, dried overnight, and stored at −80°C. Then the retina sections were performed with immunofluorescence staining of Iba-1 (1:300, ab178847, Abcam).

Formalin-fixed eyeballs were processed in paraffin and cut into 4-μm-thick sections. After deparaffinizing and rehydrating, paraffin sections were put into the antigen retrieval solution (10 mmol/L sodium citrate, 0.05%Tween20, pH=6.0) and boiled for 20 minutes in a microwave. Following cooling, the sections were performed with immunofluorescence staining of GFAP (1:500, ab7260, Abcam).

For immunofluorescence, retina tissue sections were incubated with 0.3% (vol/vol) Triton X-100/PBS. Slides were blocked with 5% normal goat serum for 1 hour at room temperature (RT), and incubated with primary antibodies overnight at 4°C. After washing with PBS, sections were incubated with appropriate secondary antibody conjugated to Alexa Fluor ^®^488 for 1 hour and then 40, 6-diamidino-2-phenylindole (DAPI) for 20 minutes. Sections were photographed on a laser microscope (TCS SP5, Leica Microsystems, Wetzlar, Germany).

### Hematoxylin-Eosin (H&E) Staining

The enucleated mice eyes were fixed in FAS eyeball fixative (G1109, Servicebio, Wuhan, China) for 24h at 4°C. Next, they were embedded in paraffin, cut into 5-μm-thick sagittal sections and then stored at room temperature. H&E staining of retina were performed after the sections were deparaffinized and rehydrated, respectively. The images were observed and imaged with a light microscope (Eclipse E100, Nikon Instruments, Melville, NY, USA).

### Real-Time PCR

Total RNA was extracted from the colonic and retinal tissue samples with RNA Extraction Kit (R0026; Beyotime) according to the manufacturer’s instructions. Real-time PCR was performed with a Thermo Real-Time PCR detection system using the Hieff Unicon^®^ Universal TaqMan multiplex qPCR master mix (11202ES03; YEASEN, Shanghai, China). The specific gene products were amplified using the following primer pairs: ZO-1: 5-GATAGCCCTGCAGCCAAAGA-3; 5-ACAATGCGGCGATAAACGTC-3; Occludin: 5-ATGTCCGGCCGATGCTCTC-3; 5-TTTGGCTGCTCTTGGGTCTGTAT-3; TLR2: 5-AAGGAGGTGCGGACTGTTTC-3, 5-CCTCTGAGATTTGACGCTTTGTC-3; TLR4: 5-TCCCTGCATAGAGGTAGTTCC-3, 5-TCAAGGGGTTGAAGCTCAGA-3; GAPDH: 5-TGTGAACGGATTTGGCCGTA-3, and 5- GTCTCGCTCCTGGAAGATGG-3. Data were analyzed according to the 2(–ΔΔCT) method.

### Western Blot Analysis

Isolated retina was extracted in cold RIPA lysis buffer composed of a protease and phosphatase inhibitor cocktail. Equal amounts of protein samples were subjected to electrophoresis on 8% or 10% tris-glycine SDS polyacrylamide gel and then transferred to a PVDF membrane. Specific primary antibodies (anti-TLR4, 1:1000, catalog no. sc-293072, Santa Cruz Biotechnology, Dallas, TX, USA; anti-NF-kB p65, 1:1000, catalog no. 8242, Cell Signaling Technology, Danvers, MA, USA; anti-p-NF-kB p65, 1:1000, catalog no. 3033, Cell Signaling Technology; anti-JNK, 1:1000, catalog no. 9252, Cell Signaling Technology; anti-p-JNK, 1:1000, catalog no. 4668, Cell Signaling Technology; anti-ZO-1, 1:1000, catalog no. 40-2200, ThermoFisher, MA, USA; anti-Occludin, 1:500, catalog no. 71-1500, Thermofisher; anti-GAPDH, 1:3000, catalog no. HC301-01, TransGen Biotech, Beijing, China) and secondary antibodies (HRP-conjugated goat anti-rabbit IgG or HRP-conjugated goat anti-mouse IgG) were used. The protein bands were detected using a commercial imaging system (ChemiScope 6300; Clinx Science Instrument Co. Ltd, Shanghai, China).

### Gut Microbiota Analysis

Total community genomic DNA from fecal samples was extracted using an E.Z.N.A. Stool DNA Kit (D4015; Omega, Dallas, Texas, USA) as the manufacturer’s instructions. The variable regions 3–4 of the 16S rRNA gene were amplified using a forward primer Nobar_341F (5′- CCTACGGGNGGCWGCAG-3′) and reverse primer Nobar_805R (5′- GACTACHVGGGTATCTAATCC-3′). All PCR reactions were carried out in 30μL reactions with 15μL of KAPA HiFi Hot Start Ready Mix (2x), 2μL of forward and reverse primers (1μM), and 2μL microbial DNA (10 ng/μL) (Takara Bio Inc., Shiga, Japan). The PCR protocol was: denaturation at 95°C for 3 min and 5 cycles of 95°C for 30 s, annealing at 45°C for 30 s, elongation at 72°C for 30 s, then 20 cycles of denaturing at 95°C for 30 s, annealing at 55°C for 30 s, elongation at 72°C for 30 s and a final extension at 72°C for 5 min. PCR products were purified by AMPure XP beads (Beckman Coulter Genomics, Danvers, USA) and quantified by Qubit2.0 (Invitrogen, Carlsbad, CA, USA). Before sequencing, the quantity of the amplicon library was assessed on Agilent 2100 Bioanalyzer (Agilent, CA, USA). Sequencing was performed using the Illumina MiSeq system (Illumina MiSeq, San Diego, CA, USA), according to the manufacturer’s instructions. 16S rRNA gene sequences were analyzed on an Illumina Miseq™/Hiseq™ platform. Paired-end reads were assigned to samples based on their unique barcode and truncated by cutting of the barcode and primer sequence. The paired-end reads were combined and clustered into operational taxonomic units (OTUs) at 97% sequence similarity using USEARCH (v7.0.1090). Representative sequences were chosen for each OUT, and taxonomic ranks were assigned to representative sequences using the Ribosomal database project (RDP) Naive Bayesian Classifier (v.2.2). A representative OUT for each group was visualized in R (v.3.6.0) using a Venn diagram. Alpha diversity including Shannon and Simpson indices was calculated using mothur v1.43.0. Beta diversity was visualized using principal component analysis (PCA) and hierarchical clustering in R (v.3.6.0). Taxonomic phylum and family relative abundances from different samples were summarized in histograms. Clustering based on the similarity between samples or microbiota communities was expressed using a heat map generated in the ‘gplots’ package for R (v3.6.0).

### Statistical Analysis

Statistical analysis was performed with SPSS 16.0.0 (SPSS Inc., Chicago, IL, USA) and GraphPad Prism 5.0 software (GraphPad Software, Inc., San Diego, CA, USA). The normality of the data was evaluated using Q-Q plots. Differences among the groups were analyzed using one-way ANOVA followed by Tukey’s *post hoc* test. The data are expressed as the mean ± SEM. A value of p < 0.05 was considered as statistically significant. Pearman’s correlation analysis was performed to evaluate the correlations between serum LPS and a series of inflammatory cytokines in serum and retina, as well as the correlations between gut microbiota and its metabolite, respectively.

## Results

### Effect of Fenofibrate on Lipid Levels of High-Fat Diet (HFD)-Induced Mice

The body weight and fat/lipid levels of mice were shown in **Table 1**. HFD fed-induced significantly increased the body weight and the fat tissue/body weight ratio of mice, compared with the SD group. Fenofibrate supplementation significantly reduced these indicators of HFD-fed mice. Compared with the SD group, the levels of total triglyceride (TG), total cholesterol (TC) and low-density lipoprotein/very low-density lipoprotein cholesterol (LDL/VLDL-C) were significantly increased, but high-density lipoprotein cholesterol (HDL-C) level was similar in the HFD group. Dietary HFD mice treated with fenofibrate showed significant reductions in TG, TC and LDL/VLDL-C levels, but no difference in HDL-C level, compared to those in the HFD group. These data revealed that fenofibrate supplementation significantly prevented and fought HFD-induced obesity and dyslipidemia.

**Table 1:** Fat/lipid levels in serum, retina and feces.

Indicators		A. SD	B. HFD	C. SD_ Fe	D. HFD_ Fe	P value		
						A versus B	A versus C	B versus D
Body weight (g)		27 ± 0.46	36 ± 0.69	25 ± 0.44	29 ± 0.60	<0.001	>0.05	<0.001
Fat tissue (% of body weight)		0.50 ± 0.05	0.96 ± 0.12	0.39 ± 0.04	0.68 ± 0.04	<0.001	>0.05	<0.05
**Serum lipid**								
TG (mmol/L)		0.26 ± 0.03	0.97 ± 0.19	0.16 ± 0.02	0.34 ± 0.03	<0.001	>0.05	<0.001
TC (μg/μL)		0.90 ± 0.06	1.77 ± 0.08	0.97 ± 0.04	1.26 ± 0.04	<0.001	>0.05	<0.001
HDL-C (μg/μL)		0.72 ± 0.04	0.75 ± 0.08	0.84 ± 0.06	0.82 ± 0.09	>0.05	>0.05	>0.05
LDL/VLDL-C (μg/μL)		0.16 ± 0.02	0.41 ± 0.03	0.13 ± 0.01	0.25 ± 0.01	<0.001	>0.05	<0.001
Total SCFAs (ng/mL)		23040 ± 2715	10674 ± 1218	18082 ± 2779	18754 ± 1482	<0.01	>0.05	<0.05
Acetic acid (ng/mL)		19561 ± 2783	7979 ± 1 004	15884 ± 2218	16440 ± 1434	<0.01	>0.05	<0.05
Propionic acid (ng/mL)		1365 ± 244.5	624.1 ± 18.42	1143 ± 241.5	895.1 ± 91.13	<0.01	>0.05	<0.05
Butyric acid (ng/mL)		382.5 ± 22.92	209.6 ± 30.55	458.4 ± 50.04	368.6 ± 39.67	<0.05	>0.05	<0.05
**Retinal lipid**								
Total SCFAs(ng/g)		26109 ± 111.7	21729 ± 26.20	29160 ± 45.44	23226 ± 80.74	<0.001	<0.001	<0.001
Acetic acid(ng/g)		25567 ± 108.2	21275 ± 21.92	28578 ± 44.46	22694 ± 83.05	<0.001	<0.001	<0.001
Propionic acid(ng/g)		224.3 ± 1.05	141.0 ± 1.97	343.3 ± 1.29	153.8 ± 1.59	<0.001	<0.001	<0.01
Butyric acid(ng/g)		27.27 ± 0.88	20.91 ± 0.67	27.34 ± 1.14	25.39 ± 0.56	<0.01	>0.05	<0.05
**Fecal lipid**								
Total SCFAs (μg/g)		1681 ± 98.59	632.5 ± 77.58	2016 ± 193.2	2128 ± 122.7	<0.001	>0.05	<0.001
Acetic acid (μg/g)		1281 ± 88.20	457.3 ± 76.99	1752 ± 178.9	1700 ± 164.3	<0.01	>0.05	<0.001
Propionic acid (μg/g)		270.2 ± 61.15	71.67 ± 11.05	171.0 ± 8.80	195.7 ± 10.04	<0.001	>0.05	<0.05
Butyric acid (μg/g)		91.34 ± 17.75	57.29 ± 8.64	46.00 ± 6.35	197.1 ± 43.06	>0.05	>0.05	<0.01

Data were expressed as mean values ± SEM. TG, Total triglyceride; TC, Total cholesterol; HDL-C, High-density lipoprotein cholesteroll LDL/VLDL-C, Low-density lipoprotein cholesterol or very low-density lipoprotein cholesterol; SCFAs, Short-chain fatty acids; SD group, standard diet group; HFD group, high-fat diet group; SD_ Fe group, standard diet plus fenofibrate group; HFD_ Fe group, high-fat diet plus fenofibrate group.

We also detected short-chain fatty acids (SCFAs) levels in serum, retina and feces (**Table 1**). Acetic acid, propionic acid and butyric acid mainly account for 90–95% of the total SCFAs (Cummings et al., 1987). The results showed that HFD significantly decreased the levels of acetic acid, propionic acid, butyric acid and total SCFAs in serum and retina, which were mitigated by fenofibrate supplementation. Similarly, the fecal levels of acetic acid, propionic acid and total SCFAs were markedly decreased, whereas butyric acid was slightly reduced in the HFD group compared to those in the SD group. Fenofibrate supplementation significantly increased the concentrations of acetic acid, propionic acid, butyric acid and total SCFAs in the feces of HFD-fed mice. Collectively, these data supported that feeding fenofibrate to HFD-induced mice significantly attenuated the reduction of SCFAs in serum, retina and feces.

### Fenofibrate Improved Intestinal Barrier Function in HFD-Fed Mice

**Figure 1:**
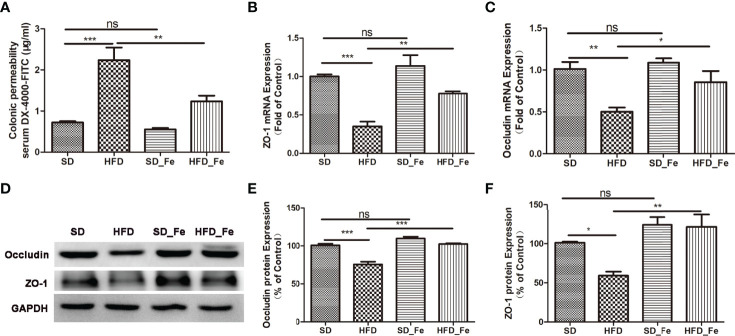
Fenofibrate improved intestinal barrier function in high-fat diet (HFD)-fed mice. **(A)** Colonic permeability assay: serum DX-4000-FITC (μg/mL). **(B, C)** ZO-1 **(B)** and Occludin **(C)** mRNA expression levels in colonic tissue. **(D–F)** Representative images and statistical analysis of Western blot showed the Occludin **(D, E)** and ZO-1 **(D, F)** protein levels in colonic tissue. SD group, standard diet group; HFD group, high-fat diet group; SD_ Fe group, standard diet plus fenofibrate group; HFD_ Fe group, high-fat diet plus fenofibrate group. n = 5; ns, not significant; ^*^P < 0.05; ^**^P < 0.01; ^***^P < 0.001. Bar graphs represent mean values ± SEM.

To investigate the effect of fenofibrate on intestinal barrier function in HFD-fed mice, we detected gut permeability using DX-4000 FITC (FD4), and the expression of epithelial tight junction proteins such as ZO-1 and Occludin. We found that HFD dramatically increased colonic permeability, and fenofibrate supplementation could inhibit this effect (**Figure 1A**). Real-time PCR and Western blot analysis showed that HFD feeding significantly reduced the expression of ZO-1 and Occludin in the colonic tissue (**Figures 1B–F**). Fenofibrate coadministration alleviated the reduction of ZO-1 and Occludin expression in the colonic tissue of HFD-fed mice (**Figures 1B–F**). These data suggested that fenofibrate treatment could partially reverse HFD-induced colonic barrier damage.

### Fenofibrate Reduced Systemic Inflammation in HFD Mice

**Figure 2:**
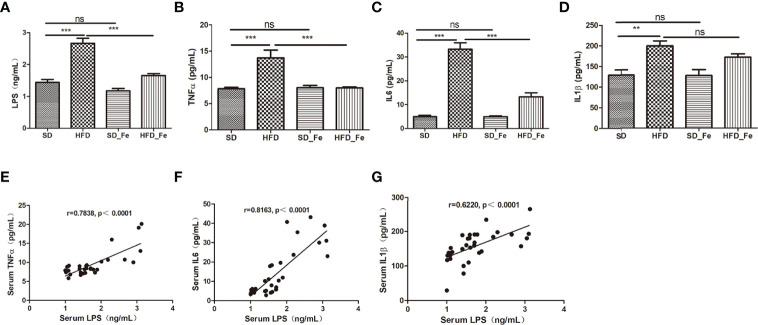
Effect of fenofibrate on inflammatory factors in the serum of high-fat diet (HFD)-fed mice. **(A–D)** Serum LPS **(A)**, TNFα **(B)**, IL6 **(C)** and IL1β **(D)** levels in the four groups. **(E–G)** Correlations between LPS and inflammatory factors TNFα **(E)**, IL6 **(F)** and IL1β **(G)** in the serum. When correlation coefficient “|r|” ≥ 0.349 (n = 32) and p < 0.05, there were significant correlations between the two indicators. SD group, standard diet group; HFD group, high-fat diet group; SD_ Fe group, standard diet plus fenofibrate group; HFD_ Fe group, high-fat diet plus fenofibrate group. n = 8 **(A–D)**; ns, not significant; ^**^P < 0.01; ^***^P < 0.001. Bar graphs represent mean values ± SEM.

To investigate the effect of dietary fenofibrate on systemic inflammation in HFD mice, the levels of serum proinflammatory cytokines including lipopolysaccharide (LPS), TNFα, IL1β, and IL6 were measured, respectively. Compared with the SD group, the levels of serum proinflammatory cytokines were increased in the HFD group (**Figures 2A–D**). Fenofibrate treatment reduced the serum levels of LPS, TNFα and IL6 in the HFD-fed mice (**Figures 2A–C**). Moreover, LPS (also referred to as endotoxin) is a potent trigger of the inflammatory response both *in vitro* and vivo. Previous studies have shown that intraperitoneal or intravenous injection of high doses of LPS into animals induces the systemic production of systemic inffammatory cytokines (Rothe et al., 1993; Li et al., 1995; Chai et al., 1996). Therefore, we further analyzed the correlations among LPS and a series of inflammatory cytokines. LPS was positively correlated with TNFα, IL6 and IL1β, respectively (**Figures 2E–G**). Collectively, our data indicated that fenofibrate alleviated the systemic inflammation in HFD-fed mice.

### Effect of Fenofibrate on the Inflammatory Response of the Retina In HFD-Induced Mice

**Figure 3:**
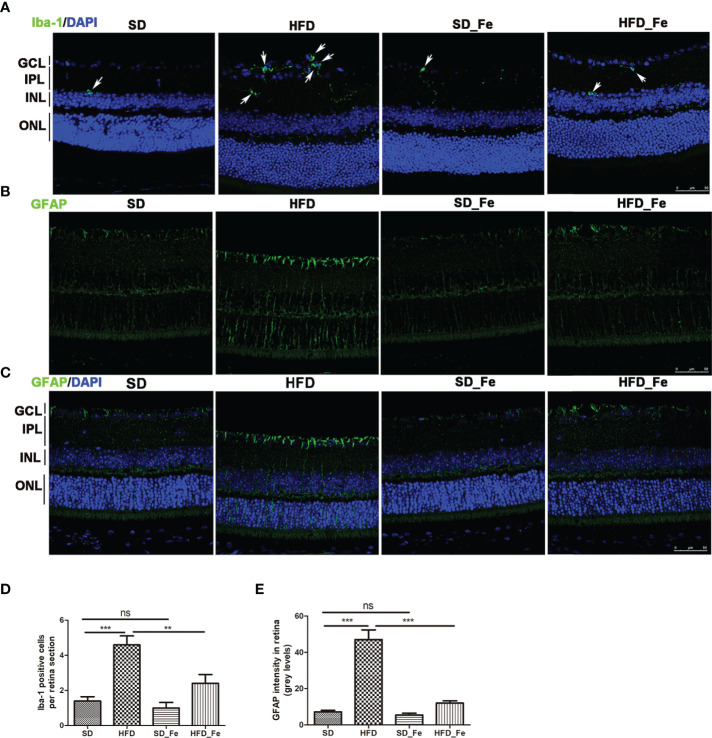
Effect of fenofibrate on the microglia and Müller cells in the retina of high-fat diet (HFD)-fed mice. **(A)** Representative images of immunofluorescence staining of Iba-1 showed microglia cells in retina (green: Iba-1, blue: DAPI). The white arrows indicated positive staining of Iba-1. **(B, C)** Representative images of immunofluorescence staining of GFAP (a Müller glial activation marker) in retina (green: GFAP, blue: DAPI). **(D)** Quantification of Iba-1 positive cell. **(E)** The fluorescent intensity of GFAP in retina. Scale bars: 50μm **(A–C)**. SD group, standard diet group; HFD group, high-fat diet group; SD_ Fe group, standard diet plus fenofibrate group; HFD_ Fe group, high-fat diet plus fenofibrate group. GCL, ganglion cell layer, IPL, inner plexiform layer, INL, inner nuclear layer, ONL, outer nuclear layer. n = 5; ns, not significant; ^**^P < 0.01; ^***^P < 0.001. Bar graphs represent mean values ± SEM.

Hematoxylin and eosin (H&E) staining showed that HFD and (or) fenofibrate supplementation did not induce dramatic histological changes on the retinas (**Supplementary Figure 1**). To further investigate the effect of fenofibrate on retinal inflammatory response in HFD-fed mice, we evaluated inflammatory cells infiltration, and toll-like receptor (TLR) 2/4 expression, NF-kB and JNK signaling pathway activation and inflammatory factors levels in retina. Microglia (Iba-1+ cells) are retinal resident macrophages originated from primitive progenitor cells in the yolk sac (Prinz et al., 2014). Upon activation by injury, microglia can become inflammatory effector cells and produce inflammatory cytokines. Müller cells are the main and unique cells of the retina, and they are the significant sources of numerous factors including inflammatory modulators. The feeding of HFD significantly increased Iba-1 positive microglia infiltration in retina (**Figures 3A, D**). Fenofibrate supplementation significantly arrested the microglia infiltration in the retina of HFD-fed mice (**Figures 3A, D**). In addition, we assessed the Müller cells activation in retina by analyzing the immunoreactivity for glial fibrillary acidic protein (GFAP). Retinal GFAP staining was stronger in the HFD group than in the SD and HFD_ Fe group (**Figures 3B, C, E**). The GFAP staining was only observed in the ganglion cell layer (GCL) of the SD and HFD_ Fe group (**Figures 3B, C**). However, the GFAP staining was detected from the GCL to the outer nuclear layer (ONL) in the retina of mice after HFD feeding for 5 months (**Figures 3B, C**). Our results suggested that fenofibrate treatment alleviated HFD-induced infiltration and activation of microglia and Müller cell in retina.

**Figure 4:**
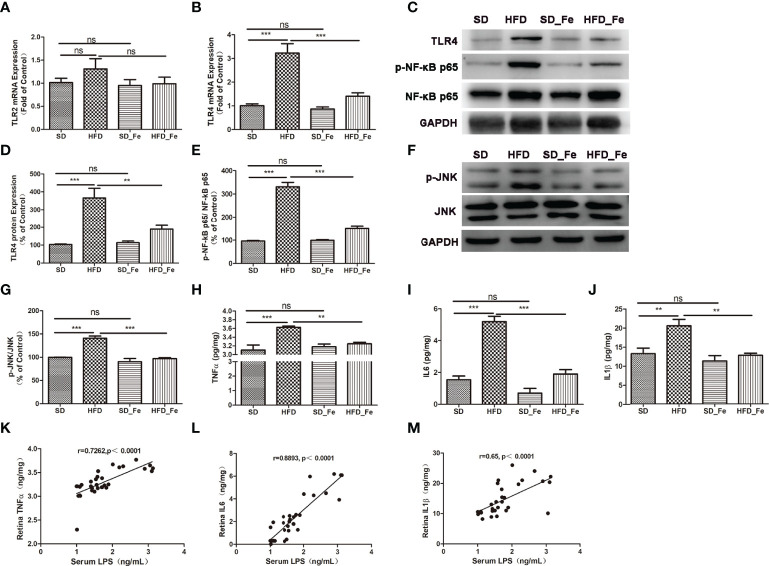
Effect of fenofibrate on the TLR2/4 signaling pathway and inflammatory factors in the retina of high-fat diet (HFD)-fed mice. **(A, B)** TLR2 **(A)** and TLR4 **(B)** mRNA expression levels in retina. **(C–E)** Representative images **(C)** and statistical analysis of Western blot showed the TLR4 protein levels **(D)** and the ratio of p-NF-kB p65/NF-kB p65 **(E)** in retina. **(F, G)** Representative images **(F)** and statistical analysis **(G)** of Western blot showed the ratio of p-JNK/JNK in the retina. **(H–J)** Inflammatory factors TNFα **(H)**, IL6 **(I)** and IL1β **(J)** levels in retina. **(K–M)** Correlations between serum LPS and TNFα **(K)**, IL6 **(L)** and IL1β **(M)** in retina, respectively. When correlation coefficient “|r|” ≥ 0.349 (n = 32) and p < 0.05, there were significant correlations between the two indicators. SD group, standard diet group; HFD group, high-fat diet group; SD_ Fe group, standard diet plus fenofibrate group; HFD_ Fe group, high-fat diet plus fenofibrate group. n = 5 **(A–G)**, n = 8 **(H–M)**; ns, not significant; ^**^P < 0.01; ^***^P < 0.001. Bar graphs represent mean values ± SEM.

TLRs recognize pathogen-associated molecular patterns (PAMPs) and then initiate proinflammatory signaling pathways (Kawai and Akira, 2010). Our results showed fenofibrate markedly inhibited TLR4 expression, but didn’t affect TLR2 level in the retina of HFD-fed mice (**Figures 4A–D**). Western blot analysis showed that HFD-fed upregulated the ratio of p-NF-kB P65/NF-kB P65 and p-JNK/JNK, and fenofibrate treatment inhibited these effects (**Figures 4C, E–G**). The inflammatory factors such as TNFα, IL1β and IL6 were significantly increased in the retina of mice after HFD feeding, and fenofibrate supplementation reversed the upregulated expressions of inflammatory cytokines (**Figures 4H–J**). These results suggested that fenofibrate suppressed inflammatory cells infiltration, TLR4 expression, NF-kB and JNK signaling pathways activation and inflammatory factors expression in the retina of HFD-fed mice.

Furthermore, we further analyzed the correlations between serum LPS and a series of inflammatory indicators in retina. Our results showed that the serum level of LPS was positively correlated with retinal TNFα, IL6 and IL1β (**Figures 4K–M**) levels, respectively.

### Fenofibrate Altered Overall Structure of Gut Microbiota of HFD-Fed Mice

**Figure 5:**
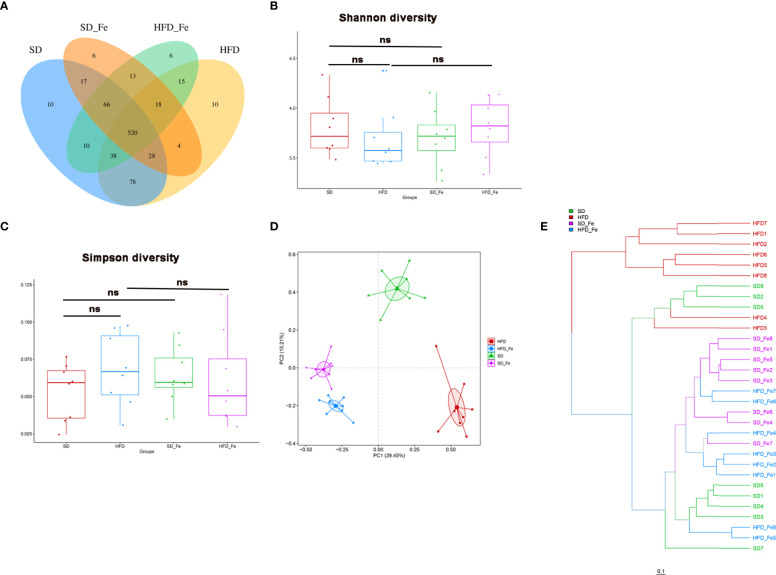
Fenofibrate altered overall structure of gut microbiota of high-fat diet (HFD)-fed mice. **(A)** Venn diagram showing the unique and shared OTUs in the different groups. **(B–C)** Community diversity of gut microbiota in mice; **(B)** Shannon diversity, **(C)** Simpson diversity. **(D)** Principal component analysis (PCA) analysis of gut microbiota in mice. **(E)** Hierarchical clustering (weight unifrac distance) of microbiota of the four groups. SD group, standard diet group; HFD group, high-fat diet group; SD_ Fe group, standard diet plus fenofibrate group; HFD_ Fe group, high-fat diet plus fenofib rate group. n = 8; ns, not significant.

To further assess whether fenofibrate administration was associated with modulation of gut microbiota composition in HFD-induced mice, the fecal samples were analyzed by 16S rRNA high throughput sequencing. After the high-throughput pyrosequencing, the 1,903,826 clean sequences were generated across all samples, with an average length of 421.125bp. These sequences were clustered into OTUs based on a 97% similarity degree. The OTU richness in each group was compared (**Figure 5A**). There were 520 OTUs shared among the SD, HFD, SD_ Fe and HFD_ Fe groups. Meanwhile, 664 (81.57%) of 814 OTUs were shared between the HFD and SD groups, and 591 (73.33%) of 806 OTUs were shared between HFD and HFD_ Fe groups.

Bacterial community diversity, quantified by Shannon and Simpson indices, was slightly lower (P>0.05) in the HFD group than those in the SD and the HFD_ Fe groups (**Figures 5B, C**). Beta-diversity for analyzing the overall composition of bacterial community was assessed by principal component analysis (PCA). OTU relative abundance analysis was performed among all the groups. The PCA score plot showed that the four groups had distinct bacterial communities (**Figure 5D**). Hierarchical clustering analysis showed that the HFD group (except the samples HFD3/4) and the other three groups revealed a clear difference in gut microbiota structure (**Figure 5E**). Compared with the HFD group, the other three groups (HFD_ Fe, SD and SD_ Fe groups) shared higher similarity in their gut microbiota structure though obvious differences still existed (**Figure 5E**). These results indicated that supplementing fenofibrate could alter overall structure of gut microbiota of HFD-fed mice.

### Fenofibrate Regulated Gut Microbiome Composition of HFD-Fed Mice

**Figure 6:**
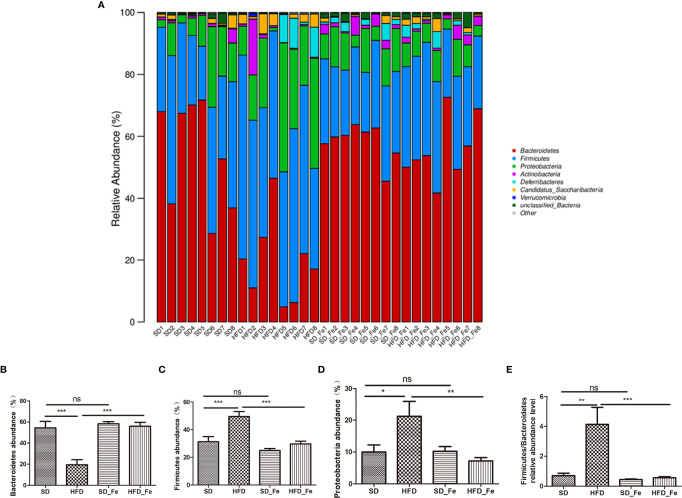
Effects of fenofibrate on the relative abundance of gut microbiota in high-fat diet (HFD) mice at the phylum level. **(A)** The relative abundance of gut microbiota at the phylum level. **(B–H)** The relative abundance of *Bacteroidetes* **(B)**, *Firmicutes* **(C)**, *Proteobacteria* **(D)** and *Firmicutes/Bacteroidetes* **(E)**. SD group, standard diet group; HFD group, high-fat diet group; SD_ Fe group, standard diet plus fenofibrate group; HFD_ Fe group, high-fat diet plus fenofibrate group. n = 8; ns, not significant; ^*^P < 0.05; ^**^P < 0.01; ^***^P < 0.01. Bar graphs represent mean values ± SEM.

The relative abundance of microbial composition in the four groups was compared at the phylum, family and genus levels. At the phylum level, the microbiome composition was dominated by *Bacteroidetes*, *Firmicutes*, *Proteobacteria*, *Actinobacteria*, *Deferribacteres* and *Candidatus_ Saccharibacteria* (**Figure 6A**). Compared with the SD group, the relative abundance of *Bacteroidetes* was significantly decreased in the HFD group, whereas the decreasing trend was significantly prevented by fenofibrate treatment (**Figure 6B**). The relative abundances of *Firmicutes* and *Proteobacteria* and the ratio of *Firmicutes*/*Bacteroidetes* were significantly increased in the HFD group, which were reversed by fenofibrate treatment (**Figures 6C–E**).

At the family level, *Porphyromonadaceae*, *Lachnospiraceae*, *Lactobacillaceae*, *Erysipelotrichaceae*, *Desulfovibrionaceae* and *Ruminococcaceae* were the primary population of gut microbiota (**Supplementary Figure 2A**). The relative abundance of *Porphyromonadaceae* was significantly decreased in the HFD group, whereas the decreasing trend was significantly prevented by fenofibrate treatment (**Supplementary Figure 2B**). The relative abundances of *Desulfovibrionaceae*, *Lachnospiraceae* and *Ruminococcacaea* were markedly higher in the HFD group compared to those in the SD and HFD_ Fe group (**Supplementary Figures 2C–E**).

Subsequently, we analyzed the gut microbiota at the genus level. Longitudinal clustering indicated the HFD group (except sample HFD4) was significantly different from the other three groups in the microbial composition at the genus level (**Supplementary Figure 3A**). As shown in **Supplementary Figures 3B–F**, compared with the SD group, the relative abundances of *unclassified_Porphyromonadaceae* (P<0.001), *Barnesiella* (P>0.05), *Alloprevotella* (P<0.05), *Parabacteroides* (P<0.001) and *Bifidobacterium* (P>0.05) were decreased in the HFD group, which were significantly reversed by fenofibrate supplementation (except *Parabacteroides*). The relative abundances of *unclassified_Desulfovibrionaceae*, *Acetatifactor*, *Flavonifractor*, *Oscillibacter* and *Anaerotruncue* were increased in the HFD group, whereas fenofibrate treatment ameliorated the above-mentioned indices in the HFD group (**Supplementary Figures 3G–J, L**). HFD feeding also induced a higher abundance of *Clostridium_XlVa* genus (**Supplementary Figure 3K**). Collectively, the results indicated that fenofibrate supplementation could modulate the gut microbial composition in HFD-fed mice.

Correlation analysis was then performed to identify association between the gut microbiota and metabolites (**Supplementary Table S1**). The results showed that *Firmicutes*, *Proteobacteria*, *Firmicutes/Bacteroidetes*, *Desulfovibrionaceae*, *unclassified_Desulfovibrionaceae*, *Acetatifactor*, *Flavonifractor*, *Oscillibacter*, *Clostridium_XlVa* and *Anaerotruncus* were positive correlation with serum LPS. *Bacteroidetes*, *Porphyromonadaceae*, *unclassified_Porphyromonadaceae*, *Barnesiella*, *Alloprovella* and *Bifidobacterium* were positively correlated with fecal SCFAs.

## Discussion

As a lipid-lowering drug, fenofibrate has been reported to have an anti-inflammatory effect in system and retina (Belfort et al., 2010; Krysiak et al., 2011; Abcouwer, 2013). However, it remained unclear about the effect of fenofibrate on HFD alone-induced systemic and retinal inflammation and its association with gut microbiota modulation. In the current study, after supplementation with fenofibrate, there were obvious improvements in dyslipidemia, SCFAs dysregulation, gut barrier dysfunction, systemic and retinal inflammation and gut microbiota dysbiosis in HFD-fed mice.

SCFAs is important in not only maintaining intestinal barrier integrity but also regulation of immune function (Cleophas et al., 2016; Parada Venegas et al., 2019). Recent study suggested that intra-peritoneal injected SCFAs can cross the blood-eye barrier and reduce intraocular inflammation induced by LPS (Chen et al., 2021). However, it was unclear about the critical role of SCFAs in the anti-inflammation action of fenofibrate. In the current study, we linked SCFAs to the inflammatory regulation of fenofibrate. Our results demonstrated that fenofibrate treatment prevented HFD-induced decreases in fecal SCFAs, accompanying with restoration of gut barrier damage and inhibition of systemic inflammation. The restoration of gut barrier helped prevent LPS translocation into systemic circulation and endotoxemia. Meanwhile, our study found that fenofibrate supplementation significantly increased the levels of SCFAs including acetic acid, propionic acid and butyric acid in the serum and retina of HFD-fed mice. Collectively, the anti-inflammation action of fenofibrate might be partially through following two mechanisms: 1. the upregulation of fecal SCFAs resulted from the fenofibrate treatment helped promote gut barrier restoration in HFD-induced mice, which prevented the leakage of LPS into circulation and LPS-associated systemic inflammation; 2. the upregulation of serum and retinal SCFAs directly inhibited local inflammation in HFD-fed mice after fenofibrate treatment.

Systemic LPS administration is commonly used as a model of neuroinflammation (Ho et al., 2015; Krishna et al., 2016; Zakaria et al., 2017). Chronic systemic inflammation damaged the blood-retina barrier, resulting in the breach of retinal immune privilege leading to the development of retinopathy (Chen and Xu, 2015). Microglia and Müller cells expressed various pattern recognition receptors (PRRs), including TLRs, and may predispose for PAMPs of intestinal origin (such as LPS) to affect retinal inflammation (Kumar et al., 2013; Lin et al., 2013; Chen and Xu, 2015). Previous study demonstrated that short-term HFD did not significantly induce the activation of microglia and Müller cells in retina of C57BL/6J mice (Kutsyr et al., 2021). Inconsistently, our results showed that long-term (5 months) HFD alone induced the upregulations of microglia and Müller cells infiltration in retina. However, fenofibrate coadministration inhibited HFD-induced inflammatory cells infiltration in retina. Among the 13 members of the TLRs family, TLR4 could recognize gram-negative LPS, while TLR2 along with TLR1 or TLR6 recognize gram-positive organism-derived PAMPs (Kawai and Akira, 2010). TLR4 signaling activation leads to the NF-kB and JNK phosphorylation, ultimately leading to chemokine and proinflammatory cytokine production (Janssens and Beyaert, 2003; Lee et al., 2019b). The current results showed that fenofibrate treatment markedly suppressed HFD-induced upregulation of TLR4 expression, NF-kB and JNK signal pathways activation and inflammatory factors levels in retina. More importantly, the positive correlation of serum LPS levels with IL1β, IL6 and TNFα in retina. Taken together, these results suggested that fenofibrate prevented LPS-associated inflammation in the retina of HFD-induced mice. Based on the above results, we speculated that fenofibrate could attenuate HFD-induced inflammation in retina, in part, through suppressing LPS-TLR4/inflammatory cells-activation of the NF-kB and JNK signaling pathways-inflammatory cytokines.

As the critical gut microbiota metabolites, SCFAs and LPS were the links between gut microbiota and host homeostasis (Burcelin et al., 2012; Arnolds and Lozupone, 2016). Previous studies reported that acetate and propionate are the main products of *Bacteroidetes* (Macfarlane and Macfarlane, 2003; Louis and Flint, 2017). The abundance of *Bacteroidetes* was decreased after HFD feeding. The predominant family *Porphyromonadaceae* which mainly included *unclassified_Porphyromonadaceae*, *Barnesiella* and *Parabacteroides* genera, as well as *Bifidobacterium* genus were associated with SCFA-production (Macfarlane and Macfarlane, 2003; Wang et al., 2019). The current study showed that HFD feeding markedly reduced the abundance of *Porphyromonadaceae* family, especially *unclassified_Porphyromonadaceae* and *Parabacteroides* genera. Moreover, the HFD-induced reduction of *Alloprevotella* genus might lead to the decrease of acetate acid and propionic acid concentrations (Qu et al., 2017). Fenofibrate coadministration significantly increased the above bacteria (except *Parabacteroides*) which were positively correlated with fecal SCFAs. The main butyrate producing-bacteria belong to the phylum *Firmicutes*, in particular the members of family *Ruminococcaceae* and *Lachnospiraceae* (Chen and Xu, 2015; Louis and Flint, 2017). This was inconsistent with decreased *Firmicutes* phylum (*Ruminococcaceae* and *Lachnospiraceae* family) and increased butyric acid in the HFD_ Fe group compared to those in the HFD group. Therefore, the mechanism of increased butyric acid in HFD mice after fenofibrate supplementation needs to be further investigated. Collectively, our results first observed that fenofibrate supplementation significantly promoted the growth of SCFAs-associated bacteria in HFD mice. Thus, we referred that the significant increase in SCFAs-associated bacteria resulted from fenofibrate treatment helped upregulate the SCFAs production, which ameliorated inflammatory response in system and retina.

Growing evidences suggested the ratio of the phyla *Firmicutes*/*Bacteroidetes* and the relative abundance of *Proteobacteria* were associated with the production of LPS, which have been proposed to promote chronic inflammatory diseases (Forte et al., 2020; Lin et al., 2020). In the current study, fenofibrate administration reduced the abundance of LPS-producing *Proteobacteria* and the ratio of the phyla *Firmicutes*/*Bacteroidetes* in the HFD-fed mice. The prominent *Desulfovibrionaceae* family in the HFD group, which mainly included *unclassified_Desulfovibrionaceae* genus exerted a proinflammatory effect (Zhang et al., 2010). Furthermore, the HFD-induced increased abundances of genera *Acetatifactor*, *Flavonifractor*, *Oscillibacter*, *Clostridium_XlVa* and *Anaerotruncus* were associated with inflammatory diseases (Conley et al., 2016; Zhang et al., 2018; Coello et al., 2019; Lee et al., 2019a; Wu et al., 2019). Following treatment with fenofibrate, the abundances of the above proinflammatory bacteria (except *Clostridium_XlVa*) were significantly decreased in the feces of HFD-fed mice. Correlation analysis revealed that LPS was positively correlated with gut microbiota which was induced by HFD and ameliorated by fenofibrate coadministration. Taken together, our findings first demonstrated that fenofibrate treatment could decrease the levels of LPS-associated bacteria in HFD-fed mice. Therefore, another possible mechanism of anti-inflammation action of fenofibrate was likely *via* decreasing the LPS-associated bacteria and subsequently reducing the systemic LPS levels, which further prevented LPS-associated inflammation in circulation and retina.

There are some limitations in the present study. Firstly, we didn’t control the daily food intake and water intake of mice. Secondly, the mice could be considered to have a similar gut microbiota composition due to the common living environment and similar dietary structure before the study. However, it will be more rigorous if gut microbiota composition of the four groups was also analyzed at the beginning of the study. Finally, fecal microbiota transplantation would be needed to further confirm the gut microbiota as a target of fenofibrate in suppressing HFD-induced retinal inflammation.

## Conclusions

The results of this study suggest that oral fenofibrate treatment has ameliorative effects on systemic and retinal inflammation in HFD mice through a mechanism that might be associated with beneficial changes in the gut microbiota/metabolites. This study provides novel insights into the mechanism of fenofibrate’s anti-inflammatory role and its association with the modulation of gut microbiota.

## Data Availability Statement

The datasets presented in this study can be found in online repositories. The names of the repository/repositories and accession number(s) can be found below: https://www.ncbi.nlm.nih.gov/, PRJNA780234.

## Ethics Statement

The animal study was reviewed and approved by Experimental animal ethics committee of Suzhou Institute of Biomedical Engineering Technology, Chinese Academy of Sciences.

## Author Contributions

XW and WL designed the experiments, XW, CY, XL, JY, YF, YW and YX conducted the experiments and analyzed the experimental data, XW and CY. drafted the manuscript. WL and YZ revised the manuscript. All authors contributed to the article and approved the submitted version.

## Funding

This work was supported by Clinical-Basic Joint PI Research Project of Aier Eye Institute (LCERI-001 [WL]).

## Conflict of Interest

The authors declare that the research was conducted in the absence of any commercial or financial relationships that could be construed as a potential conflict of interest.

## Publisher’s Note

All claims expressed in this article are solely those of the authors and do not necessarily represent those of their affiliated organizations, or those of the publisher, the editors and the reviewers. Any product that may be evaluated in this article, or claim that may be made by its manufacturer, is not guaranteed or endorsed by the publisher.
