# Supplementary material for: Fenofibrate Ameliorated Systemic and Retinal Inflammation and Modulated Gut Microbiota in High-Fat Diet-Induced Mice
Source: Front Cell Infect Microbiol. 2022 Jun 2;12:839592. doi: 10.3389/fcimb.2022.839592 (PMC9201033; doi:10.3389/fcimb.2022.839592)

Supplementary Figure 1. Effect of fenofibrate and (or) high-fat diet (HFD) on the histopathological changes of retina. (A) Hematoxylin and eosin (H&E) staining was performed in the four groups. SD group: standard diet group; HFD group: high-fat diet group; SD_ Fe group: standard diet plus fenofibrate group; HFD_ Fe group: high-fat diet plus fenofibrate group. GCL: ganglion cell layer, IPL: inner plexiform layer, INL: inner nuclear layer, OPL: outer plexiform layer, ONL: outer nuclear layer. n=5. Scale bars: 50μm.

Supplementary Figure 1

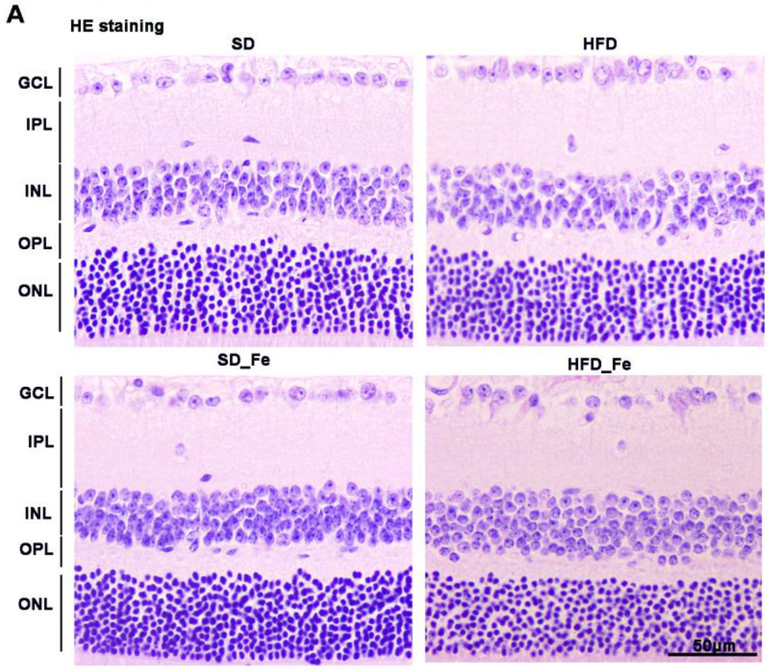

Supplement: Supplementary file 1 [file Image_1.pdf]
