# Supplementary material for: Fenofibrate Ameliorated Systemic and Retinal Inflammation and Modulated Gut Microbiota in High-Fat Diet-Induced Mice
Source: Front Cell Infect Microbiol. 2022 Jun 2;12:839592. doi: 10.3389/fcimb.2022.839592 (PMC9201033; doi:10.3389/fcimb.2022.839592)

Supplementary Figure 2. Effects of fenofibrate on the relative abundance of gut microbiota in high-fat diet (HFD) mice at the family level. (A) The relative abundance of gut microbiota at the family level. (B-E) The relative abundance of *Porphyromonadaceae* (B), *Desulfovibrionaceae* (C), *Lachnospiraceae* (D) and *Ruminococcaceae* (E). SD group: standard diet group; HFD group: high-fat diet group; SD_ Fe group: standard diet plus fenofibrate group; HFD_ Fe group: high-fat diet plus fenofibrate group. n=8; ns: not significant; $^{*}P < 0.05$; $^{**}P < 0.01$; $^{***}P < 0.01$. Bar graphs represent mean values ± SEM.

Supplementary Figure 2

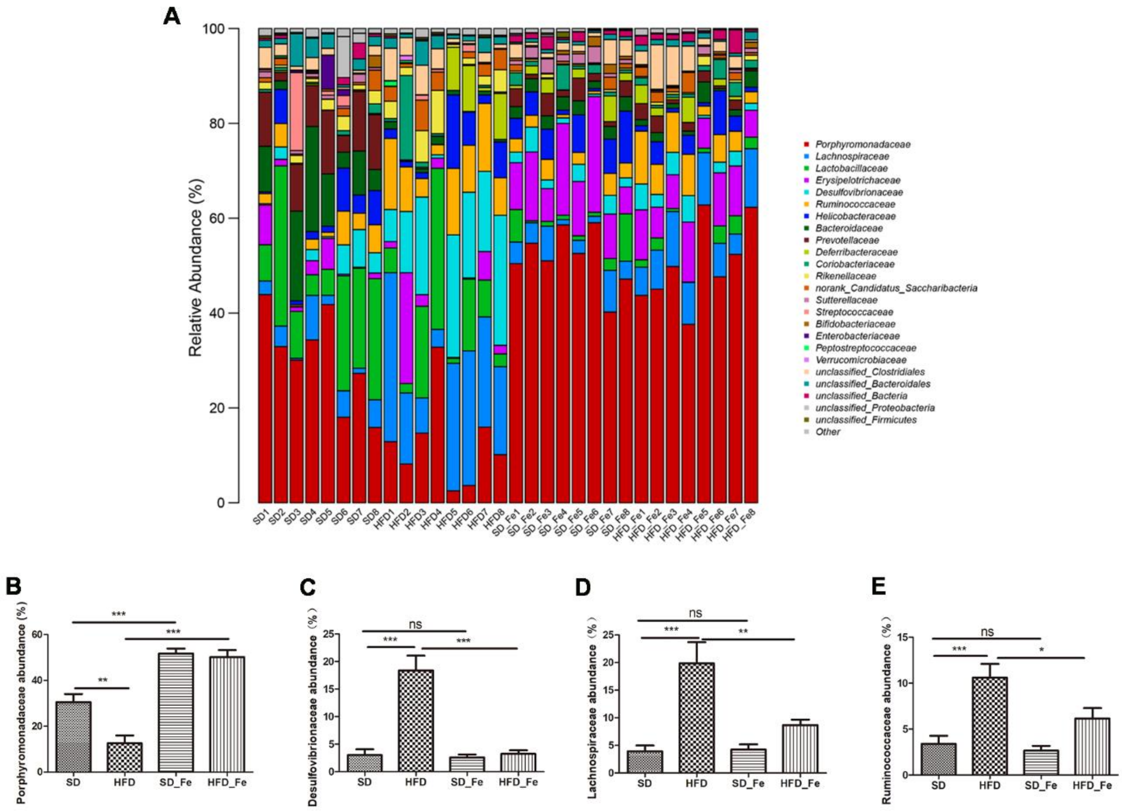

Supplement: Supplementary file 2 [file Image_2.pdf]
