# Supplementary material for: Fenofibrate Ameliorated Systemic and Retinal Inflammation and Modulated Gut Microbiota in High-Fat Diet-Induced Mice
Source: Front Cell Infect Microbiol. 2022 Jun 2;12:839592. doi: 10.3389/fcimb.2022.839592 (PMC9201033; doi:10.3389/fcimb.2022.839592)
Supplement: Supplementary file 4 [file Table_1.docx]

Supplementary Table S1. Correlations between gut microbiota and its metabolites [short-chain fatty acids (SCFAs) and lipopolysaccharide (LPS)].

| Taxa | LPS | Acetic acid | Propionic acid | Butyrate acid | Total SCFAs |
| --- | --- | --- | --- | --- | --- |
| *Bacteroidetes* | -.731^**^ | .653^**^ | .598^**^ | .110 | .614^**^ |
| *Firmicutes* | .733^**^ | -.666^**^ | -.554^*^ | -.079 | -.736^**^ |
| *Proteobacteria* | .434^*^ | -.684^**^ | -.586^*^ | -.307 | -.678^*^ |
| *Firmicutes/Bacteroidetes* | .763^**^ | -.717^**^ | -.571^*^ | -.260 | -.746^**^ |
| *Porphyromonadaceae* | -.667^**^ | .841^**^ | .448 | .204 | .828^**^ |
| *Desulfovibrionaceae* | .725^**^ | -.765^**^ | -.691^**^ | -.122 | -.792^**^ |
| *unclassified_Porphyromonadaceae* | -.551^**^ | .831^**^ | .574^*^ | .440 | .858^**^ |
| *Barnesiella* | -.673^**^ | .637^**^ | .145 | -.175 | .574^*^ |
| *Alloprovella* | -.679^**^ | .607^**^ | .340 | .281 | .607^**^ |
| *Parabacteroides* | -.662^**^ | .314 | .742^**^ | -.095 | .352 |
| *unclassified_Desulfovibrionaceae* | .680^**^ | -.705^**^ | -.655^**^ | -.170 | -.711^**^ |
| *Acetatifactor* | .689^**^ | -.603^**^ | -.432 | -.158 | -.606^**^ |
| *Flavonifractor* | .625^**^ | -.502^*^ | -.444 | -.016 | -.505^*^ |
| *Oscillibacter* | .593^**^ | -.727^**^ | -.616^**^ | -.068 | -.739^**^ |
| *Clostridium_XlVa* | .538^**^ | -.521^*^ | -.589^*^ | -.049 | -.553^*^ |
| *Anaerotruncus* | .637^**^ | -.667^**^ | -.621^**^ | -.324 | -.720^**^ |
| *Bifidobacterium* | -.298 | .634^**^ | .322 | .676^**^ | .631^**^ |

^*^P＜0.05; ^**^P＜0.01
