# Supplementary material for: Fenofibrate Ameliorated Systemic and Retinal Inflammation and Modulated Gut Microbiota in High-Fat Diet-Induced Mice
Source: Front Cell Infect Microbiol. 2022 Jun 2;12:839592. doi: 10.3389/fcimb.2022.839592 (PMC9201033; doi:10.3389/fcimb.2022.839592)

Supplementary Figure 3. Effects of fenofibrate on the relative abundance of gut microbiota in high-fat diet (HFD) mice at the genus level. (A) Comparisons of gut microbiota at the genus level on a heatmap. Longitudinal clustering indicates similarity between different samples; horizontal clustering indicates similarity of a certain species between different samples. (B-L) The relative abundance of *unclassified_Porphyromonadaceae* (B), *Barnesiella* (C), *Alloprevotella* (D), *Parabacteroides* (E), *Bifidobacterium* (F) *unclassified_Desulfovibrionaceae* (G), *Acetatifactor* (H), *Flavonifractor* (I), *Oscillibacter* (J), *Clostridium_XlVa* (K) and *Anaerotruncus* (L). SD group: standard diet group; HFD group: high-fat diet group; SD_ Fe group: standard diet plus fenofibrate group; HFD_ Fe group: high-fat diet plus fenofibrate group. n=8; ns: not significant; $^{*}P<0.05$; $^{**}P<0.01$; $^{***}P<0.001$. Bar graphs represent mean values ± SEM.

Supplementary Figure 3

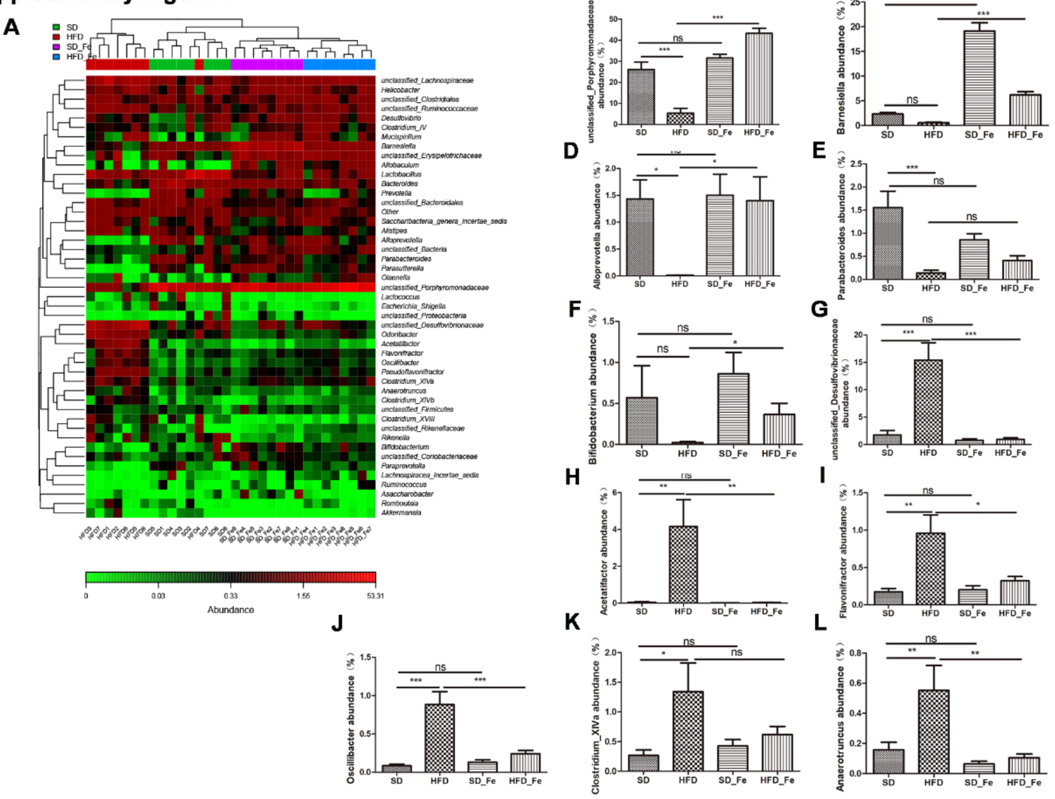

Supplement: Supplementary file 3 [file Image_3.pdf]
